# Atypical presentation of Arts syndrome due to a novel hemizygous loss-of-function variant in the *PRPS1* gene

**DOI:** 10.1016/j.ymgmr.2020.100677

**Published:** 2020-11-18

**Authors:** Sanna Puusepp, Karit Reinson, Sander Pajusalu, André B.P. van Kuilenburg, Doreen Dobritzsch, Jeroen Roelofsen, Werner Stenzel, Katrin Õunap

**Affiliations:** a Department of Clinical Genetics, United Laboratories, Tartu University Hospital, Tartu, Estonia; b Department of Clinical Genetics, Institute of Clinical Medicine, Faculty of Medicine, University of Tartu, Tartu, Estonia; c Department of Genetics, Yale University School of Medicine, New Haven, CT, USA; d Department of Clinical Chemistry, Cancer Center Amsterdam, Amsterdam Gastroenterology & Metabolism, Amsterdam UMC, University of Amsterdam, Amsterdam, the Netherlands; e Department of Chemistry, BMC, Uppsala University, Uppsala, Sweden; f Department of Neuropathology, Charité–Universitätsmedizin Berlin, Corporate Member of Freie Universität Berlin, Humboldt-Universität zu Berlin, and Berlin Institute of Health, Berlin, Germany; g Leibniz Science Campus Chronic Inflammation, Berlin, Germany

**Keywords:** *PRPS1*, Arts syndrome, Purines, Autophagy

## Abstract

The *PRPS1* gene, located on Xq22.3, encodes phosphoribosyl-pyrophosphate synthetase (PRPS), a key enzyme in de novo purine synthesis. Three clinical phenotypes are associated with loss-of-function *PRPS1* variants and decreased PRPS activity: Arts syndrome (OMIM: 301835), Charcot–Marie–Tooth disease type 5 (CMTX5, OMIM: 311070), and nonsyndromic X-linked deafness (DFN2, OMIM: 304500). Hearing loss is present in all cases. CMTX5 patients also show peripheral neuropathy and optic atrophy. Arts syndrome includes developmental delay, intellectual disability, ataxia, and susceptibility to infections, in addition to the above three features. Gain-of-function *PRPS1* variants result in PRPS superactivity (OMIM: 300661) with hyperuricemia and gout.

We report a 6-year-old boy who presented with marked generalized muscular hypotonia, global developmental delay, lack of speech, trunk instability, exercise intolerance, hypomimic face with open mouth, oropharyngeal dysphagia, dysarthria, and frequent upper respiratory tract infections. However, his nerve conduction velocity, audiologic, and funduscopic investigations were normal. A novel hemizygous variant, c.130A > G p.(Ile44Val), was found in the *PRPS1* gene by panel sequencing. PRPS activity in erythrocytes was markedly reduced, confirming the pathogenicity of the variant. Serum uric acid and urinary purine and pyrimidine metabolite levels were normal.

In conclusion, we present a novel *PRPS1* loss-of-function variant in a patient with some clinical features of Arts syndrome, but lacking a major attribute, hearing loss, which is congenital/early-onset in all other reported Arts syndrome patients. In addition, it is important to acknowledge that normal levels of serum and urinary purine and pyrimidine metabolites do not exclude *PRPS1*-related disorders.

## Highlights

• We describe a male patient with atypical presentation of Arts syndrome.
• Our patient harbors a novel loss-of-function variant in the *PRPS1* gene.
• The purine and pyrimidine levels can be normal in patients with decreased PRPS activity.

## 1 Introduction

The *PRPS1* gene (OMIM: 311850), located on the X chromosome (Xq22.3), encodes phosphoribosyl pyrophosphate synthetase (PRPS, EC 2.7.6.1) [1]. PRPS is one of the key enzymes in human metabolism because it catalyzes the synthesis of phosphoribosyl-pyrophosphate, which is a substrate for purine and pyrimidine nucleoside and nucleotide synthesis [2,3]. Nucleotides and their derivatives have numerous essential biological functions, e.g., in transfer of energy, synthesis of nucleic acids, allosteric regulation of enzymes, cell signaling, and co-enzyme activity. Therefore, it is not surprising that defects in PRPS are associated with a spectrum of clinical manifestations. Four clinical phenotypes are associated with variants of the *PRPS1* gene: Arts syndrome (OMIM: 301835), Charcot–Marie–Tooth disease type 5 (CMTX5, OMIM: 311070), nonsyndromic X-linked deafness (DFN2, OMIM: 304500), and PRPS superactivity (OMIM: 300661). The main clinical features of Arts syndrome, which is the most severe clinical presentation, are congenital sensorineural hearing loss (SNHL), early-onset muscular hypotonia, developmental delay (DD), intellectual disability (ID), lack of speech, ataxia, visual impairment, recurrent respiratory tract infections, and peripheral neuropathy. Patients with CMTX5 present with a triad of symptoms: SNHL, optic atrophy, and peripheral neuropathy. The phenotype of PRPS superactivity includes hyperuricemia and gouty arthritis, but some patients additionally present with SNHL, muscular hypotonia, DD, ID, and ataxia.

We describe a male patient with atypical presentation of Arts syndrome and a novel *PRPS1* loss-of-function variant. Of importance, our patient did not present with a major feature of SNHL, and his serum uric acid concentration and urinary purine and pyrimidine levels were normal.

## 2 Materials and methods

### 2.1 Case report

**Fig. 1:**
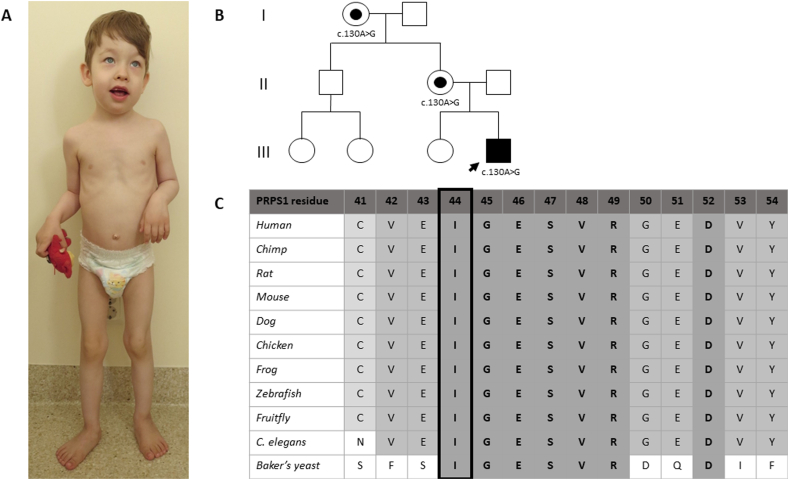
(A) The phenotype of our patient at the age of 4 years; (B) family pedigree; (C) and multialignment of the PRPS1 residues across species, showing strict conservation of Ile-44.

The patient was a boy born at term with normal birth parameters and adaptation. His neonatal otoacoustic emission screening test was normal. At the age of 2 m, his family physician noticed muscular hypotonia in the shoulder girdle. Thereafter, he developed marked generalized muscular hypotonia with decreased deep tendon reflexes and global DD. He gained head control at 7 m and started walking independently at 2y3m of age. At 3y of age, he additionally presented with considerable exercise intolerance, hypomimic face with opened mouth and pronounced saliva flow, oropharyngeal dysphagia, and dysarthria (Fig. 1A). His daily activities (e.g., walking, running, climbing the stairs, and eating) were disturbed due to trunk instability, lack of defense reflexes, and poor eye–hand coordination. Nevertheless, the strength of his limb muscles was normal. He was friendly, cooperative, and made eye contact. Based on WPPSI–IV tests, his memory functions, attention, concentration, and visual–spatial constructive skills were age-appropriate. Fine motor skills, eye–hand coordination, and receptive speech were below the normal range. Expressive speech was significantly deficient (speechless). His height (0 SD), weight (−1 SD), and occipitofrontal circumference (+2 SD) were normal, but relative macrocephaly was noted. He presented with a high forehead, bilateral epicanthus, high-arched palate, hyperplasia of gums, markedly worn teeth, pectus excavatum, and valgus feet (Fig. 1A). In addition, he had suffered from very frequent viral and bacterial upper respiratory tract and inner ear infections since the age of 4 m. At the age of 5y, he was diagnosed with epilepsy. His brain MRI investigation at 12 m of age showed small heterotopic knots of grey matter in the walls of the lateral sacs and a small arachnoid cyst. Electroneuromyography at 5 m of age was normal, but at 7 m and 12 m of age showed possible myopathic changes. Echocardiography (at 7 m of age), funduscopic examinations (at 5 m, 1y5m, and 4y2m of age), brainstem auditory evoked potential test (at 2y6m of age), and otoacoustic emission tests (at 2y9m and 4y1m of age) were normal. In infancy, acquired vitamin B12 deficiency and iron deficiency anemia were diagnosed and treated.

Mucopolysaccharides, oligosaccharides, transferrin isoelectric focusing, acid alpha-glucosidase enzyme activity, serum and urine amino acids, acylcarnitines, and very long chain fatty acids showed no significant changes. Serum organic acids showed some signs of mitochondrial disease (increased levels of lactate, pyruvate, fumarate, and malate), but the lactate/pyruvate ratio was normal. Urine organic acids also showed some signs of a defect in energy metabolism (increased levels of pyruvate, 3-OH-butyrate, sebacate, 4-OH-phenyllactate, and ketoglutarate), but other metabolites were also increased (4-OH-phenylacetate, glutarate, *N*-acetylaspartate, homovanillate, citrate, and methylmalonate). Detailed data of the organic acid analyses are shown in Supplementary tables 1 and 2.

### 2.2 Biochemistry of purine and pyrimidine metabolism

Serum uric acid was measured by an enzymatic colorimetric method (analyzer Cobas® 6000, Roche Diagnostics International Ltd.) [4]. Urinary purine and pyrimidine (PP) metabolites were measured with ultraperformance liquid chromatography–tandem mass spectrometry (UPLC–MS/MS; Waters, Milford, MA, USA), modified from Hartmann et al. (2006) [5].

### 2.3 DNA sequencing

DNA was extracted from blood lymphocytes. An expanded panel covering 4800 genes associated with monogenic disorders was sequenced (Illumina Inc., San Diego, CA, USA) and analyzed as previously described [6]. The variant was submitted to the LOVD (https://databases.lovd.nl/shared/genes/PRPS1) with variant ID 0000683199.

### 2.4 Enzyme analysis

PRPS activity was determined in a reaction mixture containing an aliquot of erythrocytes, 3 mM or 32 mM sodium phosphate, 1 mM dithiothreitol, 4.5 mM MgCl_2_, 1.0 mM ATP, 1 mM ribose-5-phosphate, and 50 mM Tris HCl (pH 7.4). The purines were removed by dilution of 100 μL frozen-packed erythrocytes with 400 μL 0.9% (*w*/*v*) NaCl and concentrated on an Amicon Ultra Ultracel 10 K Membrane filter (Millipore) by centrifugation (14,000 *g* at 4 °C for 60 min), essentially as described before [7]. Separation of AMP, ADP, and ATP was performed using a gradient from 100% to 70% 0.75 mM sodium phosphate (pH 4.55) in 20 min at a flow rate of 1.0mL/min by HPLC on an ion exchange column (Whatman PartiSphere SAX 125 mM × 4.6 mM, 5 μm particle size; VWR International, Amsterdam, the Netherlands) and a guard column (Whatman PartiSphere AX 10 mM × 2.5 mM, 5 μm particle size; VWR International) with online UV detection at 254 nm. The PRPS activity was calculated as follows: {[AMP] + ½[ADP]}/(mg protein x incubation time).

### 2.5 Molecular modeling

**Fig. 2:**
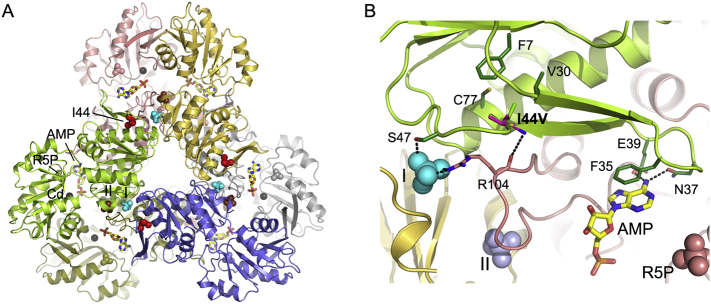
PRPS-1 structure and Ile44Val substitution site. (A) The crystal structure of PRPS-1 (PDB entry 2HCR), with each subunit colored differently. AMP (stick model colored by atom) and a Cd^2+^ ion (grey sphere) indicate the location of the ATP-Mg^2+^ binding site in the interface between the N- and C-terminal domain; the binding site for the second substrate R5P is occupied by a sulfate ion shown as a space-fill model colored as the corresponding subunit. In the crystal structure, allosteric sites I and II are also occupied by sulfate ions, shown as space-fill models in brown and cyan, respectively. The mutation site is indicated by a space-fill model of the Ile-44 side chain in red. The respective binding sites are labeled only for the green subunit. (B) Close-up view of the Ile44Val substitution site. For the valine (shown as sticks with carbon atoms in magenta), the same side chain conformer as adopted by Ile-44 (sticks with carbon atoms in green) in the crystal structure is chosen. Subunits and ligands are depicted as in (A). Residues interacting with Ile-44 or involved in ligand binding that are mentioned in the text are shown as sticks with carbon atoms in the same color as the subunit to which they belong. Hydrogen bonds are indicated as black dotted lines.

The effect of the mutation on enzyme structure was analyzed by visual inspection upon manual introduction of the altered amino acid side chain with WinCoot [8], using the crystal structure of human PRPS1 as a template (PDB-Id 2HCR) [9]. The normal mode analysis of the mutation effect was performed using the Dynamut web server (http://biosig.unimelb.edu.au/dynamut/) [10]. Fig. 2 was generated with PyMOL (http://www.pymol.org) [11].

### 2.6 Muscle biopsy analysis

At the age of 2y1m, a diagnostic muscle biopsy was performed for the right lateral vastus muscle because of initial suspicion of a congenital myopathy. Routine histological staining and enzyme histochemistry (HE, modified Gömöri trichrome, PAS, ORO, nonspecific esterase, acid phosphatase, NADH-TR, SDH, COX, and ATPase at pH 4.3 and 10.2) were performed in 8 μm cryostat sections, according to standard procedures.

As a link between *Drosophila PRPS* alleles carrying two Arts syndrome variants (Gln165Pro and Arg228Trp) and defects in autophagy processes in the *Drosophila* fat body have recently been described [12], we carried out additional immunohistochemical studies with primary antibodies against SQSTM1/p62 (Abcam, clone ab56416, dil. 1:500; Abcam, rabbit polyclonal 91526, dil. 1:100), LC3 (Nanotools Art, clone LC3-2G6, dil. 1:50), HSP70 (Abcam, clone ab6535, dil. 1:100) and BAG3 (Abcam, polyclonal, dil. 1:500). These proteins are members of chaperone-assisted selective autophagy (CASA) highlighting protein aggregations and/or increased autophagic activity in muscle tissue [13].

## 3 Results

### 3.1 Biochemistry of purine and pyrimidine metabolism

The serum uric acid level, measured at 4y of age, was 123 μmol/L (ref. 106–325 μmol/L). Urinary PP metabolites were measured twice at ages 3 and 4 years. The first PP analysis showed no pathological changes with a uric acid level of 356.2 mmol/mol creatinine (ref. 165–618 mmol/mol creatinine), hypoxanthine level of 1.339 mmol/mol creatinine (ref. 1–88.1 mmol/mol creatinine), and xanthine level of 14.3 mmol/mol creatinine (ref. 0–54.7 mmol/mol creatinine). The second analysis from a follow-up urine sample showed only traces of hypoxanthine, but uric acid and xanthine levels were normal at 348 and 17.6 mmol/mol creatinine, respectively.

### 3.2 DNA sequencing

A candidate hemizygous variant, c.130A > G p.(Ile44Val), in the *PRPS1* gene (transcript NM_002764.3) was found. The variant is located in exon 2 in the ribose-phosphate diphosphokinase domain, and is a variant of unknown significance according to the ACMG variant interpretation guidelines [14]. In silico analyses predict contradictory effects (Align-GVGD (v2007): C0; SIFT (v6.2.0): deleterious; MutationTaster (v2013): disease causing; PolyPhen-2: benign; CADD: 15.11; criterion PP3). This variant has not been reported before, and it is absent from the gnomAD database (criterion PM2). The variant was confirmed by Sanger sequencing. The patient's unaffected mother and maternal grandmother both carry this variant (Fig. 1B).

### 3.3 Enzyme analysis

PRPS activity in erythrocytes was strongly reduced to 0.08 nmol/min/mg protein (compared to controls of 0.41–1.46 nmol/min/mg protein). This functional data fulfilled the ACMG guideline criterion PS3, which allowed us to reclassify the *PRPS1* gene variant as likely pathogenic.

### 3.4 Molecular modeling

PRPS1 is a homohexameric enzyme (Fig. 2A) containing one active site and two allosteric sites (I and II) per subunit [9]. The active site is located between the N- and C-terminal domains and is primarily composed of residues from the pyrophosphate binding loop, a flexible loop, and the flag region from a neighboring subunit. The allosteric inhibitor ADP and activator phosphate bind competitively to site I, whereas phosphate binding to site II stabilizes the open conformation of the flexible active site loop, thereby promoting Mg-ATP binding. Ile-44 is strictly conserved in PRPS1 from human to yeast (Fig. 1C), and located at the end of the β-hairpin constituting the flag region, attaching it to the N-terminal domain (Fig. 2B) via hydrophobic interactions with Cys-77, Val-30, and, to a lesser extent, Phe-7. Residues Phe-35, Asn-37, and Glu-39 found at the tip of the hairpin bind substrate ATP via ring stacking or hydrogen-bonding interactions. Ser-47, located downstream of the mutation site, is involved in phosphate binding to site I, together with Arg-104 from the adjacent subunit that is hydrogen-bonded to Ile-44 via main chain atoms. The Ile44Val exchange maintains the hydrophobic character of the amino acid, but weakens its hydrophobic interactions due to the loss of a methyl group. Normal mode analysis of the protein dynamics and stability indicated that the base of the flag region will be severely destabilized, with detrimental effects on the ATP-binding site and allosteric site I architectures.

### 3.5 Muscle biopsy analysis

**Fig. 3:**
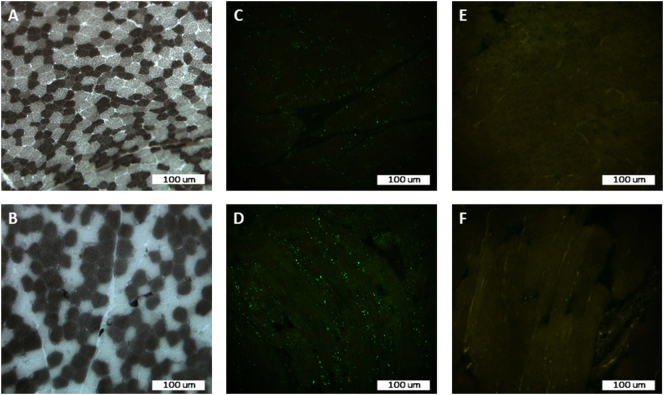
(A) ATPase at pH 10.2 and (B) ATPase at pH 4.3, showing slightly larger type 1 fibers compared to type 2 fibers; (C and D) anti- SQSTM1/p62 antibody expression in muscle fibers in transverse section (C) and in longitudinal section (D) in the patient, showing many diffuse punctae; (E and F) and anti- SQSTM1/p62 antibody expression in muscle fibers in transverse section (E) and in longitudinal section (F) in a control muscle biopsy, showing single dots.

The muscle biopsy from the right lateral vastus muscle showed mild fiber size variation, with type 1 fibers being slightly larger than type 2 fibers (Fig. 3A and B). No other abnormalities were noted on routine staining and enzyme histochemistry. Reaction with the antibody against SQSTM1/p62 showed diffuse punctae in many muscle fibers representing prominent autophagosomes within the sarcoplasm, which are not present in a control muscle biopsy (Fig. 3C–F). No staining reaction was detected with anti-LC3, anti-HSP70, or anti-BAG3 antibodies.

## 4 Discussion

We describe a challenging clinical case of a boy who was initially suspected to have a congenital myopathy, however, abnormalities in urinary and serum organic acid analyses suggested a defect in energy metabolism. Surprisingly, we diagnosed an inborn error of purine metabolism: a *PRPS1*-related disorder with a novel variant in the *PRPS1* gene.

Arts et al. [15] described a five-generation family of 12 males presenting with features later referred to as Arts syndrome. These patients were confirmed to have pathogenic variants in the *PRPS1* gene by de Brouwer et al. [16] Four additional Arts syndrome families with six affected males have been reported to date [[16], [17], [7], [18]]. All 18 patients had congenital/early-onset SNHL, ID, muscular hypotonia, and muscle weakness, 17 had visual impairment, 16 had ataxia, 15 showed DD, 14 showed susceptibility to infections, 12 died in early childhood, four developed peripheral neuropathy, three presented with muscle weakness exacerbation after infections, two had seizures, and one developed behavioral disturbances. In addition, 10 male patients from four families with the classical symptom triad of CMTX5 (early-onset SNHL, peripheral neuropathy, and optic atrophy) [[19], [20], [21]], another 10 male patients from four families with only SNHL and peripheral neuropathy [[22], [23], [24]], and 36 male patients from eight families with DFN2 [23,[25], [26], [27], [28]] with variants in the *PRPS1* gene have been reported. Intrafamilial phenotypic variation was described in a Spanish family with nine affected males—all presented with prelingual severe-to-profound SNHL, but three males had additional features (global DD, mild ID, seizures, and clubfoot) [26]. Furthermore, nine male patients with hyperuricemia and gout (PRPS superactivity) also harbored distinctive point mutations in the *PRPS1* gene, and most of these patients additionally presented with DD, ID, muscular hypotonia, SNHL, peripheral neuropathy, and/or frequent infections [[29], [30], [31], [32]].

Our patient's phenotype includes some features characteristic of Arts syndrome (early-onset hypotonia, delayed psychomotor development, and susceptibility to infections), however, he has not developed two important symptoms, hearing impairment and optic atrophy. While optic atrophy has also been missing or with a later onset in some of the previously described Arts syndrome and CMTX5 patients, it is highly interesting that at the age of five years, our patient has not yet developed hearing loss, which was early-onset in all Arts syndrome, as well as CMTX5, patients. Of note, studies suggest an important role for the PRPS enzyme in the inner ear: *PRPS1* is expressed in cochlear and vestibular hair cells and spiral ganglion neurons in mice [25]. Zebrafish models with *prps1a* and *prps1b* mutants have shown reduced numbers of inner ear hair cells, which resulted in significant loss of hearing in zebrafish [33,34]. Interestingly, an increase in apoptotic activity rather than a decrease in proliferation of inner ear hair cells was suggested [34]. We hypothesize that our patient may develop hearing loss later on, or alternatively, the residual activity of the PRPS enzyme could be higher in nucleated cells than in erythrocytes. The severity of the *PRPS1*-related disorders largely correlates with the residual PRPS activity measured in erythrocytes or fibroblasts. Patients with Arts syndrome showed markedly decreased PRPS activity while DFN2 patients showed only a mild to moderate decrease in activity [[15], [16], [17], [7], [18], [19], [20],^23^,^25^,^27^]. Patients with PRPS superactivity had increased PRPS activity in fibroblasts; however, patients with additional neurological features showed a marked decrease in PRPS activity in erythrocytes [30]. Moreover, two patients with hyperuricemia and congenital SNHL, DD, and severe respiratory infections had normal PRPS activity in fibroblasts and decreased activity in erythrocytes [31]. In another patient with congenital urolithiasis and DD, the PRPS activity in erythrocytes was slightly decreased, but at a low Pi concentration, the defective PRPS had higher activity than in controls [32]. These data refer to complex metabolic consequences resulting from different variants in the *PRPS1* gene.

We identified a novel variant, c.130A > G p.(Ile44Val), in the *PRPS1* gene, and performed molecular modeling and normal mode analysis of the PRPS protein to obtain additional data for its pathogenicity since it is a great challenge to confirm novel X-linked variants [35]. We found that the exchange of Ile to Val at position 44 destabilizes the ATP binding site (Fig. 2B), which is in agreement with previous reports. In general, variants causing Arts syndrome or CMTX5 phenotype disturb the ATP binding site, DFN2 variants affect only the local structure of the protein, and gain-of-function variants disturb one or both of the allosteric regulatory sites resulting in decreased inhibition of the enzyme and PRPS superactivity [[16], [17], [7],^19^,^23^,^25^,^26^,[30], [31], [32]].

As the clinical picture of inborn errors of PP metabolism is highly diverse and can overlap with other disorders, metabolic assays for detection of biomarkers of PP metabolism are important first-line diagnostic tools [2,3]. The hallmark of PRPS superactivity is hyperuricemia. Therefore, we would expect to see hypouricemia in patients with decreased PRPS enzyme activity. However, 19 out of 24 patients tested, including our patient, had normal serum uric acid levels [17,7,19,22,[24], [25], [26], [27]], and 5 out of 24 had levels at the lower limit of the reference range [16,18,19]. Urinary PP analysis has been described in two *PRPS1* case reports, with hypoxanthine <1 mmol/mol creatinine (ref. 2–55 mmol/mol creatinine) measured by reversed-phase HPLC in two brothers [16], and a hypoxanthine value of 3 mmol/L (ref. 2–32 mmol/L), which was regarded as reduced, measured by HPLC in another patient [7]. We present the third case of a *PRPS1*-related disorder with the results of two urinary PP analyses performed with a one-year time difference and measured by UPLC–MS/MS. The first PP analysis was normal, and in the second analysis, performed because of the surprising diagnosis of a purine metabolism disorder, hypoxanthine was below our lower reference cut-off of 1 mmol/mol creatinine. However, as our laboratory's experience shows that hypoxanthine values are frequently below this cut-off value, it is debatable whether this finding should be regarded as pathological. The validation results of different PP analysis methods have shown variable reference ranges for PP metabolites [5,[36], [37], [38]], and in some studies, only the upper reference cut-off has been set for hypoxanthine along with the majority of other PP metabolites [38]. It should also be noted that serum, plasma, and urinary uric acid and other PP metabolite concentrations also depend on the patient's diet and sample preparation [[39], [40], [41]]. Hence, it is important to acknowledge that a PP analysis with no pathological changes does not exclude a *PRPS1*-related disorder.

A possible pathomechanism for the development of neurological symptoms in patients with Arts syndrome could be a dysfunction in lysosome-mediated and autophagy processes. In particular, in a *Drosophila* model of Arts syndrome engineered and studied by Delos Santos et al. [12], the mutant flies, indeed, showed locomotive defects, deregulated basal autophagy and lysosome homeostasis, and hypersensitivity to oxidative stress. In addition, accumulation of lipid droplets in pupal eyes and protein aggregation in the brains of the mutant flies were observed. We showed an upregulation of SQSTM1/p62 in our patient's muscle biopsy specimen (Fig. 3C and D), which denotes an increased autophagic flux as the p62 protein is an autophagy receptor transporting cargo to autophagosomes [13]. As the expression of LC3, BAG3 and HSP70 proteins was normal, the increase in p62 probably does not reflect increased macroautophagy or CASA-related autophagy, but may be related to microautophagy. In previously described cases, one patient with Arts syndrome and peripheral neuropathy showed neurogenic changes [18], and another patient with PRPS1 superactivity had a normal muscle biopsy result [31]. However, the authors did not mention, whether immunohistochemistry for SQSTM1/p62 was performed on these muscle biopsies or not. Further investigations regarding autophagy processes in patients with *PRPS1* variants could bring new insights.

Our patient also presented with acquired vitamin B12 deficiency in infancy. As a population-based extended newborn screening has revealed a very high incidence of congenital vitamin B12 deficiency in Estonia (33.8/100,000 live births), we believe that this feature is a coincidental finding rather than part of the Arts syndrome phenotype [42].

## 5 Conclusion

We present a patient with a novel *PRPS1* loss-of-function variant. It is clear that *PRPS1* defects result in a disease spectrum with clinical overlap between different features, rather than being four allelic disease entities. Our case, with the absence of a major feature—hearing loss—further confirms this notion. We also emphasize that serum and urinary biochemical markers of PP disorders can be, and even may typically be, normal in patients with decreased PRPS activity.

## Funding

This research was funded by 10.13039/501100002301Estonian Research Council, Estonia (grant IDs PUT355, PRG471, and PUTJD827).

## Compliance with ethical standards

This study was approved by the Research Ethics Committee of the University of Tartu, Estonia (approval date December 19, 2016 and number 265/T-12). Written informed consent for the study and for publication of the patient's photograph was obtained from the patient's mother.

## Declaration of Competing Interest

The authors declare that they have no conflict of interest.

## Appendix A Supplementary data

Supplementary tables Image 1
