# Supplementary material for: Atypical presentation of Arts syndrome due to a novel hemizygous loss-of-function variant in the PRPS1 gene
Source: Mol Genet Metab Rep. 2020 Nov 18;25:100677. doi: 10.1016/j.ymgmr.2020.100677 (PMC7689168; doi:10.1016/j.ymgmr.2020.100677)
Supplement: Supplementary file 1 — Supplementary tables [file mmc1.docx]

**Supplementary table 1.** The concentrations of serum organic acids, analysed at 3 years of age. Increased values are marked bold.

| **Compound** | **Concentration (μmol/L)** | **Reference values (μmol/L)** |
| --- | --- | --- |
| Lactate | **3891.24** | 700-3300 |
| Pyruvate | **210.48** | 27-160 |
| 2-OH-butyrate | 63.11 | 8-80 |
| 3-OH-butyrate | 91.26 | 22-700 |
| Acetoacetate | 24.32 | 0-86 |
| Benzoate | 6.56 |  |
| Succinate | 28.46 | 0-32 |
| Glycerate | 4.60 | 0-24 |
| Fumarate | **18.48** | 0-4 |
| Malate | **80.94** | 0-21 |
| Ketoglutarate | 5.22 | 0-23 |
| Citrate | 73.14 | 30-400 |

**Supplementary table 2.** The concentrations of urinary organic acids, analysed at 3 years of age. Increased values are marked bold.

| **Compound** | **Concentration (mmol/mol Cr)** | **Reference values for ages 1 month-5 years (mmol/mol Cr)** |
| --- | --- | --- |
| Lactate | 77.19 | 33 - 285 |
| Glycolate | 192.54 | 0.2 - 198 |
| Pyruvate | **44.20** | 5.1 - 22.6 |
| 2-OH-butyrate | 2.75 | 0.2 -5.1 |
| 3-OH-butyrate | **53.19** | 0 -11.1 |
| 2-OH-3-methylbutyrate | 0 | 0 -1.3 |
| Acetoacetate | 0.57 | 0.2-5.8 |
| Malonate | 0 | <2 |
| 3-OH-isovalerate | 56.94 | 10.4-67 |
| Methylmalonate | **8.88** | 0-2 |
| Benzoate | 12.01 | 0.6-7.7 |
| Ethylmalonate | 3.95 | 1.7-14.6 |
| Succinate | 26.63 | 17.6-79.2 |
| Glycerate | 5.58 | 4.2-32.2 |
| Uracil | 5.78 | 6.9-56.6 |
| Fumarate | 6.01 | 1.4-9.9 |
| Glutarate | **10.78** | 0-5.3 |
| 3-methylglutarate | 2.38 | 0 - 7 |
| 3-methylglutaconate | 13.95 | 0-19 |
| Malate | 14.59 | 2.2-16.2 |
| Adipate | 4.82 | 0-34.3 |
| 5-oxoproline | 66.11 | 25.8-92.2 |
| Mevalonate | 0 | 0.1-0.3 |
| 2-OH-glutarate | 34.94 | 5-26.8 |
| 3-OH-glutarate | 0.25 | <8 |
| 3-OH-3-methylglutarate | 38.45 | 6.2-49.7 |
| Ketoglutarate | **132.40** | 29.8-117 |
| 4-OH-phenylacetate | **177.79** | 12.3- 174 |
| N-acetylaspartate | **54.07** | 7-40.8 |
| Suberate | 1.28 | 0-10.1 |
| Orotate | 2.27 | 0.02-3.6 |
| Homovanillate | **17.52** | 4-13 |
| Azelate | 0.36 | 0-15.4 |
| Citrate | **1709.23** | 75-667 |
| Hippurate | 277.79 | 119-1390 |
| Vanillylmadelic acid | 15.19 | 0-17 |
| Sebacate | **1.67** | 0-1.4 |
| 4-OH-phenyllactate | **5.14** | 0.03-3.1 |
| Vanillyllactate | 0.07 | 0-10 |
